# Antibiotic Resistance Patterns of *Escherichia coli* Isolates from Hospitals in Kumasi, Ghana

**DOI:** 10.5402/2012/658470

**Published:** 2012-10-14

**Authors:** Duredoh Freeman George, Stephen Yao Gbedema, Christian Agyare, Francis Adu, Vivian Etsiapa Boamah, Adelaide Ama Tawiah, Sixtus Bieranye Bayaa Martin Saana

**Affiliations:** Department of Pharmaceutics, Faculty of Pharmacy and Pharmaceutical Sciences, Kwame Nkrumah University of Science and Technology, Kumasi, Ghana

## Abstract

Nosocomial infections are infections acquired by a patient as a result of treatment in a hospital or healthcare service providing center and symptoms occurs within a short period of hospitalization. The study was to determine the antibiotic resistance patterns of *Escherichia coli* isolated from Kumasi-South, Tafo and Suntreso Hospitals, Kumasi, Ghana. Total of 600 swabs samples from the hospitals were collected between January and June, 2010. The isolates were identified using morphological and biochemical means. A total of 97 *E. coli* isolates were obtained from the hospitals. Beds in hospital wards had the highest number of *E. coli* strains (53.6%), followed by floors (20.6%) while drainages had the least isolates (3.1%). Majority of the *E. coli* isolates (90.7%) exhibited resistance to ampicillin while 6.2 and 3.1% showed intermediate and sensitive respectively. Co-trimoxazole, 78.4% of the isolates were resistant while 9.3 and 12.4% exhibited intermediate and sensitive responses respectively. *E. coli* isolates (28.6 to 46.4%) were resistant to gentamicin, ciprofloxacin and ceftriaxone while 14.4 to 47.4% gave intermediate responses. Most isolates (80.4%) exhibited multi-drug resistance. There is a need to observe proper personal hygiene, use of effective disinfectants and proper disposal of contaminated/pathogenic materials in these hospitals to control nosocomial infections.

## 1. Introduction 

Nosocomial infections, also called healthcare-associated infections are those infections acquired by a patients as a result of treatment in a hospital, clinic or healthcare service centre. These infections generally appear 48 hours or more after hospital admission or within 30 days after discharge. They occur because of instrumentation, increased use of antimicrobial agents, breaks in aseptic techniques and lack of hand hygiene [1]. At any time, over 1.4 billion people worldwide suffer from infectious complications acquired in hospital [2]. Microorganisms often implicated in these infections include *Escherichia coli*, *Pseudomonas aeruginosa*, *Klebsiella* species, *Staphylococcus aureus* and *Mycobacterium tuberculosis. *According to the American National Nosocomial Infections Surveillance, more than 40% of nosocomial infections occurred in parts of Asia, Latin America, and Sub-Saharan Africa [3].

*E. coli* is facultative anaerobe and can undergo both fermentative and respiratory metabolisms. *E. coli* is nonspore-forming and beta hemolytic. It usually ferments lactose on MacConkey agar to produce pink colonies with surrounding areas of precipitated bile salts. It also presents with a green sheen on eosin methylene blue agar. *E. coli* strain will produce indole from tryptophan; it does not produce hydrogen sulfide, urease, and cannot use citrate as sole carbon source [1]. 

In some hospitals, *E. coli* ranked first as the most common cause of hospital-acquired infections [4]. *E. coli* strains were found to be the highest and most frequent among the pathogenic microorganisms isolated from ten teaching hospitals in China [5]. Pathogenic strains of *E. coli* are responsible for three types of infections in humans; urinary tracts infections, neonatal meningitis, and intestinal diseases [6].

In many West African countries, nosocomial infections are abound but not much study has been done to determine the proportion of infections acquired by patients or health workers from hospital and or healthcare-providing facilities. Hospitals serve a reservoir of various types of microorganisms; some may be multiple resistant to antibiotics [8] and the selective pressure of antimicrobial use in hospitals, therefore makes the environment a repository for these resistant strains [9]. Newman and his colleagues reported on the occurrence of nosocomial infections in Korle-Bu Teaching Hospital in Accra, Ghana [10]. These studies are therefore necessary and need to be conducted in many other parts of the country in order to generate national data on these pathogenic organisms, more especially on their antibiotic resistant patterns in Ghana. This study sought to determine the antibiotic resistance patterns of *E. coli* isolates from the premises of three hospitals in Kumasi, Ghana.

## 2. Materials and Methods

The protocols for the study were approved by the individual Hospital's Ethics Committees. The samples were collected from Kumasi South, Tafo and Suntreso Hospitals in Kumasi, Ghana. A total of 600 swabs samples of floors, benches, beds, door handles, and waste water from drainages were collected between January and June, 2010. The swabs were put into sterile test tubes, closed tightly, and labeled appropriately. All the materials including culture media, reference antibiotics, and reagents were purchased from Oxoid, Basingstoke, United Kingdom unless otherwise stated.

### 2.1. Cultivation and Isolation of *Escherichia coli* Isolates

The various samples collected were separately inoculated into 10 mL of nutrient broths and incubated at 37°C for 24 hours. Using a sterile platinum loop, each culture was separately streaked onto the surface of MacConkey Agar plates, labeled and incubated at 37°C for 48 hours, and observed for signs of growth and colony appearance. 

Colonies that appeared pink on the MacConkey agar plates were removed with sterile inoculating wire and separately streaked onto the surface of eosin methylene blue agar plates. The plates were then incubated at 37°C for 24 hours. Isolated black-colored colonies with metallic sheen were again fished out into nutrient broths and incubated at 37°C for 24 hours. The various subcultures were streaked onto nutrient agar slants, incubated at 37°C for 48 hours, and then kept in the refrigerator at −20°C for further identification and antibiotic sensitivity studies.

### 2.2. Identification and Confirmation of *Escherichia coli* Isolates

The *E. coli* isolates were screened through the various microscopic examination and biochemical reactions to confirm their identities. These included indole, oxidase, and arginine dehydrolase production, citrate utilization, nitrite reduction, fermentation of carbohydrates (such as xylose, maltose, arabinose, glycerol, and starch), methyl red-Voges Proskauer test, and reaction Triple sugar iron agar [11, 12]. All the tests were performed on reference-typed culture of *E. coli* (ATCC 25922).

### 2.3. Antibiotic Sensitivity Test

Kirby-Bauer disc diffusion method [13] as recommended by the Clinical and Laboratory Standards Institute [7] was used to determine the *in vitro* susceptibility of the identified *E. coli* isolates to gentamicin (GM) 10 *μ*g, ciprofloxacin (CIP) 5 *μ*g, ceftriaxone (CRO) 30 *μ*g, ampicillin (AMP) 10 *μ*g, and cotrimoxazole (SXT) (trimethoprim-sulphamethoxazole) 25 *μ*g. A standardized suspension of the isolated *E. coli* was prepared by inoculating a colony into 10 mL peptone water and incubated at 37°C for 24 hours. It was then diluted to 0.5 MacFarland turbidity standards. A sterile swab was dipped into the standardized inoculum and used to inoculate evenly the surface of already prepared Mueller-Hinton agar (Oxoid Basingstoke, UK). The agar was left for 15 minutes for the surface moisture to dry. A multichannel disc dispenser (Oxoid, Basingstoke, UK) was used to deposit the antibiotics discs onto the surface of the inoculated medium. The plate was then incubated at 37°C for 18 hours. The zones of growth inhibition were recorded. The method was replicated three times and the mean zones of inhibition compared with figures (Table 1) provided by the Clinical and Laboratories Standards Institute [13]. *Escherichia coli *ATCC 25922 was used as control.

## 3. Results and Discussion

**Figure 1:**
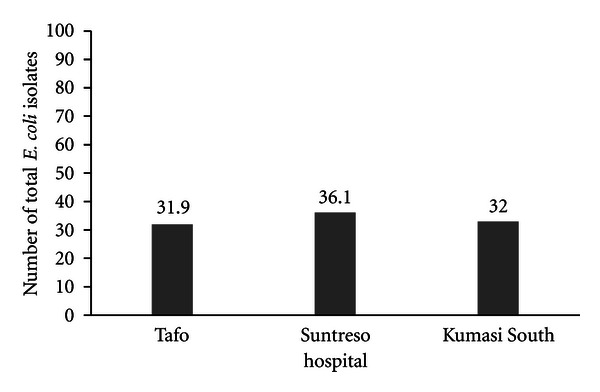
Distribution of *E. coli* isolates within the three hospitals: Kumasi South, Tafo and Suntreso hospitals, Kumasi, Ghana.

A total of 150 (lactose fermenter) isolates recovered on MacConkey agar (Oxoid, Basingstoke, UK) were suspected to be *E. coli.* These were screened through the various microscopic examination and biochemical reactions. *E. coli* isolates were identified from the various locations (benches, floor, door handles and drainages, male, female, and pediatrics wards) in the three hospitals (Figure 1). A total of 97 isolates from the three hospitals were confirmed as *E. coli*. 

Jarvis and Martone [14] reported that *E. coli* as the most common nosocomial pathogen in some hospitals in the United States. Also, *E. coli* has been reported to be among the most frequent isolates in hospitals in Ethiopia [15]. 

Among the three hospitals from which the samples or swabs were taken, *E. coli* isolates were widely distributed in various locations throughout the three hospitals for which samples were analyzed (Table 2).

**Figure 2:**
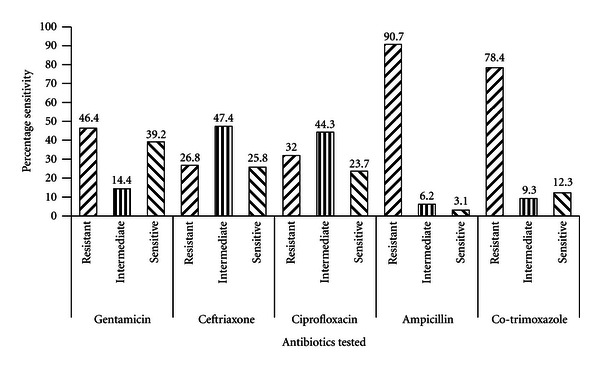
Antibiotic sensitivity patterns of *E. coli *isolates to reference antibiotics determined by Kirby-Bauer agar disc diffusion method and the mean zones of inhibition compared with the values provided by the Clinical and Laboratory Standards Institute [7].

About 90% of the *E. coli* isolates exhibited resistance to ampicillin while 6.2 and 3.1%, respectively, showed intermediate and sensitive. For cotrimoxazole (trimethoprim-sulphamethoxazole), 78.4% of the isolates were resistant while 9.3 and 12.3% intermediate and sensitive responses. Between 26.8 to 46.4% of the *E. coli* isolates also showed resistance to gentamicin, ciprofloxacin, and ceftriaxone, while 14.4 to 47.4% gave intermediate responses. Ceftriaxone, ciprofloxacin, and gentamicin sensitive isolates were also in the range of 23.7 to 39.2% (Figure 2).

The majority of the gentamicin sensitive *E. coli* isolates (28.9%) was isolated from the male wards followed by floor samples (21.1%) as shown in Table 3. None of the drainage samples were resistant to gentamicin, while 20% each from the floor and female wards proved resistant. 26.9% isolates from bench samples exhibited intermediate response to gentamicin. Out of the total, 46 *E. coli* isolates exhibited intermediate response to ceftriaxone, 30% were from the male wards, 21.4% from floor and 2.2% from drainage samples. Most of the *E. coli* resistant isolates (26.9%) were from the benches and 19.2% from male wards while no resistant strains were recovered from door handles (Table 3).

Ciprofloxacin-resistant *E. coli* isolates were recovered from floor samples (29%) followed by the samples from pediatric wards (19.4%). *E. coli* isolates which exhibited intermediate response to ciprofloxacin (30%) were in the samples/swabs from the male ward and none from door handles. Majority of the *E. coli* sensitive isolates were from female wards (30.4%) followed by male and pediatric wards samples (17.4%). Floors and benches samples showed equal ciprofloxacin sensitive *E. coli* strains of 13%. The distributions of ampicillin resistant *E. coli* isolates were 22.7, 20.5, and 19.3% for male wards, floors, and benches, respectively (Table 3). Many of the isolates obtained were found to be resistant to more than two different classes of the reference antibiotics (Table 4).

Majority of the *E. coli* isolates (53.6%) were isolated from the hospital beddings while about 21% were from floor samples (Table 2). Most of the *E. coli* isolates (90 to 78%) were resistant to ampicillin and cotrimoxazole, respectively (Figure 1). The high occurrence of *E. coli* isolates in these samples could be attributed to poor hygienic conditions in these hospitals and the overcrowding in these hospitals due to inadequate number of health care facilities in the region.

A total of 46.4, 32.0, and 26.8% of the *E. coli* isolates exhibited resistance to gentamicin, ciprofloxacin, and ceftriaxone, respectively, and these were similar to what was reported by Yismaw and his colleagues [16]. Yismaw et al. [16] also reported a similar resistance pattern of *E. coli* to gentamicin (47%), ciprofloxacin (33%), and ceftriaxone (26%). And these high levels of antibiotic resistance have been attributed to widespread abuse of these antibiotics [15]. Out of 97 *E. coli* isolates, 78 isolates or 80.4% exhibited multiple drug resistance [17] to at least three different classes of the reference antibiotics used. These high numbers of resistant *E. coli* isolates in the hospitals are potential reservoirs of resistant genes which can easily be transferred to other pathogens. Hence, there is a need to observe proper hygiene, use of effective disinfectants, and monitor the administration and prescription of antibiotics in hospitals.

## 4. Conclusion 

Most of the *E. coli* isolates (80.4%) exhibited multiple drug resistance and measures such as observation of proper personal hygiene by health staff and patients, use of effective disinfectants in reducing the possible pathogenic organisms in these hospitals, and so forth. These findings have therefore showed the need for the hospital management to be concerned about the potential of hospitalized patients becoming infected with some nosocomial infections, especially resistant strains of *E. coli*. 

## Figures and Tables

**Table 1:** Acceptable susceptible zone of inhibition values for antibiotics used based on *CLSI.

Antibiotic	Resistant	Intermediate	Susceptible
Ciprofloxacin (5 *μ*g)	≤15	16–20	≥21
Ampicillin (10 *μ*g)	≤13	14–16	≥17
Gentamicin (10 *μ*g)	≤12	13-14	≥15
Ceftriaxone (30 *μ*g)	≤13	14–20	≥21
Co-trimoxazole (25 *μ*g)	≤10	11–15	≥16

*CLSI: Clinical and Laboratory Standards Institute [7].

**Table 2:** Number of samples containing *E. coli* isolates in the swabs/samples taken from the three hospitals.

	Tafo hospital (*n*)	Kumasi South hospital (*n*)	Suntreso hospital (*n*)
Beds	17 (90)	17 (90)	18 (90)
Floors	7 (30)	3 (30)	10 (30)
Benches	5 (30)	6 (30)	6 (30)
Door handles	0 (30)	5 (30)	0 (30)
Drainages	0 (20)	3 (20)	0 (20)

Key: *n*: total number of swabs/samples taken from the hospital.

**Table 3:** Sources of *E. coli* isolates in relation to antibiotic sensitivity patterns as compared to with values provided by the Clinical and Laboratories Standards Institute [7].

Type of reference antibiotics and resistance patterns of *E. coli* isolates		Source of sample							Total
		Floors	Drainages	Door handles	Benches	Female wards	Male wards	Pediatric wards	
Gentamicin									
Intermediate	Count (%)	3 (21.4)	1 (7.1)	0 (0)	4 (28.6)	3 (21.4)	1 (7.1)	2 (14.3)	14 (100)
Resistant	Count (%)	9 (20.0)	0 (0)	1 (2.2)	6 (13.3)	9 (20.0)	11 (24.4)	9 (20.0)	45 (100)
Sensitive	Count (%)	8 (21.1)	2 (5.3)	1 (2.6)	7 (18.4)	6 (15.8)	11 (28.9)	3 (7.9)	38 (100)
Ceftriaxone									
Intermediate	Count (%)	12 (26.1)	1 (2.2)	2 (4.3)	4 (8.7)	8 (17.4)	14 (30.4)	5 (10.9)	46 (100)
Resistant	Count (%)	3 (11.5)	2 (7.7)	0 (0)	7 (26.9)	4 (15.4)	5 (19.2)	5 (19.2)	26 (100)
Sensitive	Count (%)	5 (20.0)	0 (0)	0 (0)	6 (24.0)	6 (24.0)	4 (16.0)	4 (16.0)	25 (100)
Ciprofloxacin									
Intermediate	Count (%)	8 (18.6)	1 (2.3)	0 (0)	10 (23.3)	5 (11.6)	15 (34.9)	4 (9.3)	43 (100)
Resistant	Count (%)	9 (29.0)	1 (3.2)	1 (3.2)	4 (12.9)	6 (19.4)	4 (12.9)	6 (19.4)	31 (100)
Sensitive	Count (%)	3 (13.0)	1 (4.3)	1 (4.3)	3 (13.0)	7 (30.4)	4 (17.4)	4 (17.4)	23 (100)
Ampicillin									
Intermediate	Count (%)	0 (0)	1 (16.7)	0 (0)	0 (0)	1 (16.7)	2 (33.3)	2 (33.3)	6 (100)
Resistant	Count (%)	18 (20.5)	2 (2.3)	2 (2.3)	17 (19.3)	17 (19.3)	20 (22.7)	12 (13.6)	88 (100)
Sensitive	Count (%)	2 (66.7)	0 (0)	0 (0)	0 (0)	0 (0)	1 (33.3)	0 (0)	3 (100)
Co-trimoxazole									
Intermediate	Count (%)	2 (22.2)	1 (11.1)	0 (0)	1 (11.1)	2 (22.2)	2 (22.2)	1 (11.1)	9 (100)
Resistant	Count (%)	14 (18.4)	1 (1.3)	2 (2.6)	15 (19.7)	14 (18.4)	17 (22.4)	13 (17.1)	76 (100)
Sensitive	Count (%)	4 (33.3)	1 (8.3)	0 (0)	1 (8.3)	2 (16.7)	4 (33.3)	0 (0)	12 (100)

**Table 4:** Multiple-drug resistant (MDR)* pattern of *E. coli* isolates determined by Kirby-Bauer agar disc diffusion method and mean zones of inhibition compared with the values provided by the Clinical and Laboratory Standards Institute [7].

Hospital	Total No. of MDR *E. coli *	Total no. of *E. coli *isolates	Percent of MDR
Tafo	27	30	90
Suntreso	28	34	82.4
Kumasi-South	23	33	69.7

Total	78	97	80.4

*Multiple drug resistance (MDR) is defined as resistance of organism (bacteria) to at least three different antibiotics.
